# Apoptosis protein markers in comorbid type 2 diabetes mellitus and depression; mediators of vascular contributions to cognitive impairment

**DOI:** 10.1002/alz70856_100566

**Published:** 2025-12-25

**Authors:** Sofia Perfetto, Myuri Ruthirakuhan, Yuen Yan Wong, Si Won Ryoo, Celine H.L. Huang, Bradley J. MacIntosh, Walter Swardfager

**Affiliations:** ^1^ University of Toronto, Toronto, ON, Canada; ^2^ Sunnybrook Research Institute, Toronto, ON, Canada

## Abstract

**Background:**

Cerebrovascular disease and apoptosis are independently associated with an increased risk of dementia, for which depression and type 2 diabetes mellitus (T2DM) are risk factors. This study investigated whether the association between concentrations of apoptosis‐related proteins and cerebrovascular MRI markers differ by T2DM and depression status.

**Method:**

We used a cross‐sectional cohort from the UK Biobank participants and stratified by: neither T2DM nor depression; depression only; T2DM only; T2DM with depression. Depression was accessed from ICD‐10 records or a physician diagnosis of major depression. T2DM was ascertained as ICD‐10 status, physician diagnosis of T2DM, glycated hemoglobin>=48mmol/mol, or based on use of an antidiabetic medication. Concentrations of apoptosis‐related proteins (B‐cell lymphoma‐2 [BCL‐2]; BCL‐2‐associated X protein [BAX]; caspase‐3) were quantified from peripheral blood plasma by antibody‐based Olink Explore 3072 Proximity Extension Assay. White matter fractional anisotropy (FA) and mean diffusivity (MD) were quantified from diffusion tensor images. White matter hyperintensity (WMH) volumes were quantified from T2‐weighted fluid‐attenuated inversion recovery images at 3T. Linear regression models were used to evaluate associations between apoptosis protein concentrations and vascular imaging outcomes across the strata and controlling for age, sex, ethnicity, body mass index, total cholesterol, hypertension, and total intracranial volume.

**Result:**

1309 were included in the study (*N* = 1018 with neither T2DM nor depression (52% female, age=53.5±7.6 years), *N* = 102 with depression only (68% female, age=54.1±7.8 years), *N* = 161 with T2DM only (36% female, age=55.5±6.8 years), *N* = 28 with both T2DM and depression (50% female, age=53.4±8.2 years). In T2DM and depression, higher concentrations of BAX were correlated with lower FA (β=‐0.086, 95% CI=[‐0.045,‐0.011]), higher MD (β=0.066, 95% CI=[3.29×10^−6^, 3.41×10^−5^]) and greater WMH volumes (β=0.082, 95% CI=[0.002, 0.009); higher BAX‐to‐BCL2 ratio was correlated with lower FA (β=‐0.067, 95% CI=[‐0.041,‐0.004]) and higher WMH volumes (β=0.064, 95% CI=[0.082,0.009]); and higher caspase‐3 concentrations were correlated with lower FA (β=‐0.072, 95% CI=[‐0.039,‐0.006]), higher MD (β=0.057, 95% CI=[1.11×10^−6^, 3.04×10^−5^]) and greater WMH volumes (β=0.077, 95% CI=[0.002,0.009]) (all, *p* <.05).

**Conclusion:**

These findings suggest that among individuals with T2DM and depression, elevated peripheral blood apoptosis markers are associated with cerebral microvascular complications, pointing to unique pathophysiological elements underlying mood and metabolic comorbidity.

